# Multinational characterization of neurological phenotypes in patients hospitalized with COVID-19

**DOI:** 10.1038/s41598-021-99481-9

**Published:** 2021-10-12

**Authors:** Trang T. Le, Alba Gutiérrez-Sacristán, Jiyeon Son, Chuan Hong, Andrew M. South, Brett K. Beaulieu-Jones, Ne Hooi Will Loh, Yuan Luo, Michele Morris, Kee Yuan Ngiam, Lav P. Patel, Malarkodi J. Samayamuthu, Emily Schriver, Amelia L. M. Tan, Jason Moore, Tianxi Cai, Gilbert S. Omenn, Paul Avillach, Isaac S. Kohane, The Consortium for Clinical Characterization of COVID-19 by EHR (4CE), James R. Aaron, Giuseppe Agapito, Adem Albayrak, Mario Alessiani, Danilo F. Amendola, François Angoulvant, Li L. L. J. Anthony, Bruce J. Aronow, Andrew Atz, James Balshi, Douglas S. Bell, Antonio Bellasi, Riccardo Bellazzi, Vincent Benoit, Michele Beraghi, José Luis Bernal Sobrino, Mélodie Bernaux, Romain Bey, Alvar Blanco Martínez, Martin Boeker, Clara-Lea Bonzel, John Booth, Silvano Bosari, Florence T. Bourgeois, Robert L. Bradford, Gabriel A. Brat, Stéphane Bréant, Nicholas W. Brown, William A. Bryant, Mauro Bucalo, Anita Burgun, Mario Cannataro, Aldo Carmona, Charlotte Caucheteux, Julien Champ, Krista Chen, Jin Chen, Luca Chiovato, Lorenzo Chiudinelli, James J. Cimino, Tiago K. Colicchio, Sylvie Cormont, Sébastien Cossin, Jean B. Craig, Juan Luis Cruz Bermúdez, Jaime Cruz Rojo, Arianna Dagliati, Mohamad Daniar, Christel Daniel, Anahita Davoudi, Batsal Devkota, Julien Dubiel, Loic Esteve, Shirley Fan, Robert W. Follett, Paula S. A. Gaiolla, Thomas Ganslandt, Noelia García Barrio, Lana X. Garmire, Nils Gehlenborg, Alon Geva, Tobias Gradinger, Alexandre Gramfort, Romain Griffier, Nicolas Griffon, Olivier Grisel, David A. Hanauer, Christian Haverkamp, Bing He, Darren W. Henderson, Martin Hilka, John H. Holmes, Petar Horki, Kenneth M. Huling, Meghan R. Hutch, Richard W. Issitt, Anne Sophie Jannot, Vianney Jouhet, Ramakanth Kavuluru, Mark S. Keller, Katie Kirchoff, Jeffrey G. Klann, Ian D. Krantz, Detlef Kraska, Ashok K. Krishnamurthy, Sehi L’Yi, Judith Leblanc, Andressa R. R. Leite, Guillaume Lemaitre, Leslie Lenert, Damien Leprovost, Molei Liu, Sarah Lozano-Zahonero, Kristine E. Lynch, Sadiqa Mahmood, Sarah Maidlow, Adeline C. Makoudjou Tchendjou, Alberto Malovini, Kenneth D. Mandl, Chengsheng Mao, Anupama Maram, Patricia Martel, Aaron J. Masino, Michael E. Matheny, Thomas Maulhardt, Maria Mazzitelli, Michael T. McDuffie, Arthur Mensch, Fatima Ashraf, Marianna Milano, Marcos F. Minicucci, Bertrand Moal, Cinta Moraleda, Jeffrey S. Morris, Karyn L. Moshal, Sajad Mousavi, Douglas A. Murad, Shawn N. Murphy, Thomas P. Naughton, Antoine Neuraz, James B. Norman, Jihad Obeid, Marina P. Okoshi, Karen L. Olson, Nina Orlova, Brian D. Ostasiewski, Nathan P. Palmer, Nicolas Paris, Miguel Pedrera Jimenez, Emily R. Pfaff, Danielle Pillion, Hans U. Prokosch, Robson A. Prudente, Víctor Quirós González, Rachel B. Ramoni, Maryna Raskin, Siegbert Rieg, Gustavo Roig Domínguez, Pablo Rojo, Carlos Sáez, Elisa Salamanca, Arnaud Sandrin, Janaina C. C. Santos, Maria Savino, Juergen Schuettler, Luigia Scudeller, Neil J. Sebire, Pablo Serrano Balazote, Patricia Serre, Arnaud Serret-Larmande, Zahra Shakeri, Domenick Silvio, Piotr Sliz, Charles Sonday, Anastasia Spiridou, Bryce W. Q. Tan, Byorn W. L. Tan, Suzana E. Tanni, Deanne M. Taylor, Ana I. Terriza-Torres, Valentina Tibollo, Patric Tippmann, Carlo Torti, Enrico M. Trecarichi, Yi-Ju Tseng, Andrew K. Vallejos, Gael Varoquaux, Margaret Vella, Jill-Jênn Vie, Michele Vitacca, Kavishwar B. Wagholikar, Lemuel R. Waitman, Demian Wassermann, Griffin M. Weber, Yuan William, Nadir Yehya, Alberto Zambelli, Harrison G. Zhang, Daniela Zoeller, Chiara Zucco, Shyam Visweswaran, Danielle L. Mowery, Zongqi Xia

**Affiliations:** 1 Department of Biostatistics, Epidemiology and Informatics, University of Pennsylvania Perelman School of Medicine, Philadelphia, PA USA; 2 Department of Biomedical Informatics, Harvard Medical School, Boston, MA USA; 3 Department of Neurology, University of Pittsburgh, Biomedical Science Tower 3, Suite 7014, 3501 5th Avenue, Pittsburgh, PA 15260 USA; 4 Department of Pediatrics, Wake Forest School of Medicine, Winston Salem, NC USA; 5 Department of Critical Care, National University Health Systems, Singapore, Singapore; 6 Department of Preventive Medicine, Northwestern University, Chicago, IL USA; 7 Department of Biomedical Informatics, University of Pittsburgh, Pittsburgh, PA USA; 8 Department of Surgery, National University Health Systems, Singapore, Singapore; 9 Department of Internal Medicine, University of Kansas Medical Center, Kansas City, KS USA; 10 Data Analytics Center, University of Pennsylvania Health System, Philadelphia, PA USA; 11 Department of Computational Medicine and Bioinformatics, University of Michigan, Ann Arbor, MI USA; 12 Department of Biomedical Informatics, University of Kentucky, Lexington, KY USA; 13 Department of Legal, Economic and Social Sciences, University Magna Graecia of Catanzaro, Catanzaro, Italy; 14 Health Catalyst, INC., Cambridge, MA USA; 15 Department of Surgery, ASST Pavia, Lombardia Region Health System, Pavia, Italy; 16 Clinical Research Unit of Botucatu Medical School, São Paulo State University, Botucatu, Brazil; 17 Pediatric Emergency Department, Hôpital Necker-Enfants Malades, Assistance Publique-Hôpitaux de Paris, Paris, France; 18 National Center for Infectious Diseases, Tan Tock Seng Hospital, Singapore, Singapore; 19 Departments of Biomedical Informatics, Pediatrics, Cincinnati Children’s Hospital Medical Center, University of Cincinnati, Cincinnati, OH USA; 20 Department of Pediatrics, Medical University of South Carolina, Charleston, SC USA; 21 Department of Surgery, St. Luke’s University Health Network, Bethlehem, PA USA; 22 Department of Medicine, David Geffen School of Medicine at UCLA, Los Angeles, CA USA; 23 UOC Ricerca, Innovazione e Brand Reputation, ASST Papa Giovanni XXIII, Bergamo, Italy; 24 Department of Electrical, Computer and Biomedical Engineering, University of Pavia, Pavia, Italy; 25 IT Department, Innovation & Data, APHP Greater Paris University Hospital, Paris, France; 26 I.T. Department, ASST Pavia, Pavia, Italy; 27 Health Informatics, Hospital Universitario, 12 de Octubre, Madrid, Spain; 28 Strategy and Transformation Department, APHP Greater Paris University Hospital, Paris, France; 29 Faculty of Medicine and Medical Center, University of Freiburg, Freiburg, Germany; 30 Digital Research, Informatics and Virtual Environments (DRIVE), Great Ormond Street Hospital for Children, London, UK; 31 Scientific Direction, IRCCS Ca’ Granda Ospedale Maggiore Policlinico di Milano, Milan, Italy; 32 North Carolina Translational and Clinical Sciences (NC TraCS) Institute, University of North Carolina, Chapel Hill, NC USA; 33 BIOMERIS (BIOMedical Research Informatics Solutions), Pavia, Italy; 34 Department of Biomedical Informatics, HEGP, APHP Greater Paris University Hospital, Paris, France; 35 Department of Medical and Surgical Sciences, Data Analytics Research Center, University Magna Graecia of Catanzaro, Catanzaro, Italy; 36 Department of Anesthesia, St. Luke’s University Health Network, Bethlehem, PA USA; 37 Université Paris-Saclay, Inria, CEA, Palaiseau, France; 38 INRIA Sophia-Antipolis–ZENITH Team, LIRMM, Montpellier, France; 39 Computational Health Informatics Program, Boston Children’s Hospital, Boston, MA USA; 40 Department of Internal Medicine, University of Kentucky, Lexington, KY USA; 41 Unit of Internal Medicine and Endocrinology, Istituti Clinici Scientifici Maugeri SpA SB IRCCS, Pavia, Italy; 42 Department of Internal Medicine and Therapeutics, University of Pavia, Pavia, Italy; 43 Informatics Institute, University of Alabama at Birmingham, Birmingham, AL USA; 44 IAM Unit, Bordeaux University Hospital/ERIAS-Inserm U1219 BPH, Bordeaux, France; 45 Biomedical Informatics Center, Medical University of South Carolina, Charleston, SC USA; 46 Clinical Research Informatics, Boston Children’s Hospital, Boston, MA USA; 47 Department of Biomedical and Health Informatics, Children’s Hospital of Philadelphia, Philadelphia, PA USA; 48 SED/SIERRA, Inria Centre de Paris, Paris, France; 49 Health Information Technology & Services, University of Michigan, Ann Arbor, MI USA; 50 Internal Medicine Department, Botucatu Medical School, São Paulo State University, Botucatu, Brazil; 51 Heinrich-Lanz-Center for Digital Health, University Medicine Mannheim, Heidelberg University, Mannheim, Germany; 52 Department of Anesthesiology, Critical Care, and Pain Medicine Boston Children’s Hospital, Boston, MA USA; 53 Department of Learning Health Sciences, University of Michigan Medical School, Ann Arbor, MI USA; 54 MSHI Medical University of South Carolina, Charleston, SC USA; 55 Department of Medicine, Massachusetts General Hospital, Boston, MA USA; 56 Division of Human Genetics, Department of Pediatrics, The Children’s Hospital of Philadelphia, Philadelphia, PA USA; 57 Center for Medical Information and Communication Technology, University Hospital, Erlangen, Germany; 58 Renaissance Computing Institute/Department of Computer Science, University of North Carolina, Chapel Hill, NC USA; 59 Clinical Research Unit, Saint Antoine Hospital, APHP Greater Paris University Hospital, Paris, France; 60 Clevy.io, Paris, France; 61 Department of Biostatistics, Harvard T.H. Chan School of Public Health, Boston, MA USA; 62 VA Informatics and Computing Infrastructure, VA Salt Lake City Health Care System, Salt Lake City, USA; 63 MICHR Informatics, University of Michigan, Ann Arbor, MI USA; 64 Laboratory of Informatics and Systems Engineering for Clinical Research, Istituti Clinici Scientifici Maugeri SpA SB IRCCS, Pavia, Italy; 65 Harvard Catalyst, Harvard Medical School, Boston, MA USA; 66 Clinical Research Unit, Paris Saclay, APHP Greater Paris University Hospital, Paris, France; 67 Department of Anesthesiology and Critical Care, Children’s Hospital of Philadelphia, Philadelphia, PA USA; 68 VA Informatics and Computing Infrastructure, Tennessee Valley Healthcare System, Veterans Affairs Medical Center, Washington, D.C., USA; 69 École Normale Supérieure, PSL Université Paris, Paris, France; 70 BIG-ARC, The University of Texas Health Science Center at Houston, School of Biomedical Informatics, Houston, TX USA; 71 Pediatric Infectious Disease Department, Hospital Universitario, 12 de Octubre, Madrid, Spain; 72 Department of Infectious Diseases, Great Ormond Street Hospital for Children, London, UK; 73 Department of Neurology, Massachusetts General Hospital, Boston, MA USA; 74 Internal Medicine Department of Botucatu Medical School, São Paulo State University, Botucatu, Brazil; 75 Department of Pediatrics, Boston Children’s Hospital, Boston, MA USA; 76 Center for Biomedical Informatics, Wake Forest School of Medicine, Winston-Salem, NC USA; 77 Department of Medical Informatics, University of Erlangen-Nürnberg, Erlangen, Germany; 78 Department of Veterans Affairs, Office of Research and Development, Baltimore, MD USA; 79 Biomedical Data Science Lab, ITACA Institute, Universitat Politècnica de València, València, Spain; 80 Nurse Department of FMB-Medicine School of Botucatu, Botucatu, Brazil; 81 Management Engineering, ASST Pavia, Lombardia Region Health System, Pavia, Italy; 82 Department of Anesthesiology, University Hospital Erlangen, FAU Erlangen-Nürnberg, Erlangen, Germany; 83 Critical Care Medicine, Department of Medicine, St. Luke’s University Health Network, Bethlehem, PA USA; 84 Department of Medicine, National University Hospital, Singapore, Singapore; 85 Department of Information Management, Chang Gung University, Taoyuan, Taiwan; 86 Clinical & Translational Science Institute, Medical College of Wisconsin, Milwaukee, WI USA; 87 Montréal Neurological Institute, McGill University, Montreal, Canada; 88 SequeL, Inria Lille, Villeneuve-d’Ascq, France; 89 Respiratory Department, ICS S Maugeri IRCCS, Pavia, Italy; 90 Department of Health Management and Informatics, University of Missouri, Columbia, MO USA; 91 Department of Oncology, ASST Papa Giovanni XXIII, Bergamo, Italy

**Keywords:** Neurology, Neurological disorders, Medical research, Epidemiology

## Abstract

Neurological complications worsen outcomes in COVID-19. To define the prevalence of neurological conditions among hospitalized patients with a positive SARS-CoV-2 reverse transcription polymerase chain reaction test in geographically diverse multinational populations during early pandemic, we used electronic health records (EHR) from 338 participating hospitals across 6 countries and 3 continents (January–September 2020) for a cross-sectional analysis. We assessed the frequency of International Classification of Disease code of neurological conditions by countries, healthcare systems, time before and after admission for COVID-19 and COVID-19 severity. Among 35,177 hospitalized patients with SARS-CoV-2 infection, there was an increase in the proportion with disorders of consciousness (5.8%, 95% confidence interval [CI] 3.7–7.8%, *p*_FDR_ < 0.001) and unspecified disorders of the brain (8.1%, 5.7–10.5%, *p*_FDR_ < 0.001) when compared to the pre-admission proportion. During hospitalization, the relative risk of disorders of consciousness (22%, 19–25%), cerebrovascular diseases (24%, 13–35%), nontraumatic intracranial hemorrhage (34%, 20–50%), encephalitis and/or myelitis (37%, 17–60%) and myopathy (72%, 67–77%) were higher for patients with severe COVID-19 when compared to those who never experienced severe COVID-19. Leveraging a multinational network to capture standardized EHR data, we highlighted the increased prevalence of central and peripheral neurological phenotypes in patients hospitalized with COVID-19, particularly among those with severe disease.

## Introduction

The World Health Organization declared coronavirus disease 2019 (COVID-19) due to the severe acute respiratory syndrome coronavirus 2 (SARS-CoV-2) infection as a global pandemic on March 11, 2020^1^. Growing evidence points to the multi-organ involvement of COVID-19, particularly the nervous system, which increases morbidity and mortality^2–4^. Given the health consequences of neurological complications, recognizing the neurological phenotypes associated with COVID-19 would inform prevention, diagnosis and treatment that could potentially mitigate disability and death.

Early reports highlighted central and peripheral neurological phenotypes in adults with COVID-19, including cerebrovascular disease^5^, meningoencephalitis and encephalomyelitis^6,7^, encephalopathy^5^, cranial neuropathies^8^, Guillain-Barré syndrome^9–11^, plexopathy^12^, anosmia and ageusia^13,14^ and cognitive and neuropsychiatric issues^15^. Children with COVID-19 have similar presentations, including ischemic stroke, encephalopathy, headache, and muscle weakness^16–18^. Prior prevalence studies of neurological conditions in COVID-19 largely examined data from single countries (*e.g.,* China^19^, the United Kingdom^20,21^ and Italy^22^) or single healthcare systems^4,12,23–25^. Few *large-scale* studies have used a *standardized* data capture approach to examine the multinational prevalence of the spectrum of neurologic conditions in COVID-19 patients and with *careful local* data quality control, particularly those who experienced severe respiratory and/or critical illness status^26,27^.

Electronic health records (EHRs) data can facilitate clinical discovery efforts. Our team created the International Consortium for Clinical Characterization of COVID-19 by EHR (4CE; http://www.covidclinical.net) to standardize and aggregate multinational EHR data (from 34 healthcare systems and 338 affiliated hospitals across six countries at the time of data freeze for this study) to address critical clinical and epidemiological questions relevant to COVID-19^28–31^. Central to the 4CE effort is the ability of local clinician experts and data scientists at each contributing healthcare system to ensure the quality of common EHR data elements. Leveraging the highly scalable, federated, multinational networks of the 4CE consortium, we computed the prevalence of a wide range of central and peripheral neurological conditions in hospitalized patients with reverse transcription polymerase chain reaction (PCR)-confirmed SARS-CoV-2 infection by healthcare system and by country. We also compared the differences in the prevalence of neurological conditions between patients with and those without severe COVID-19 based on the internationally validated 4CE severity criteria^30^.

## Results

**Figure 1:**
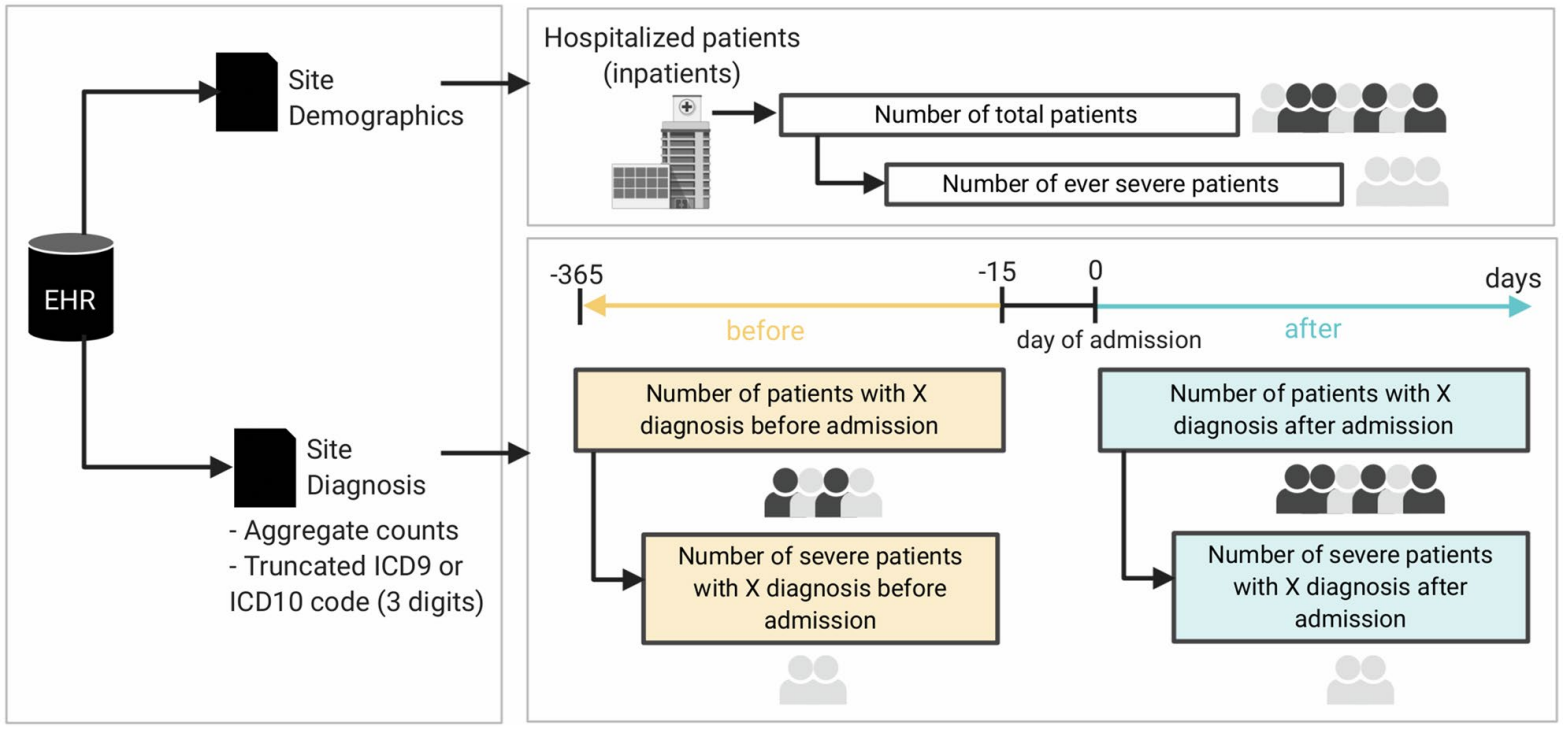
Schematic diagram of the cohort and data generation workflow for each healthcare system. The figure was created with Biorender (Biorender.com).

**Figure 2:**
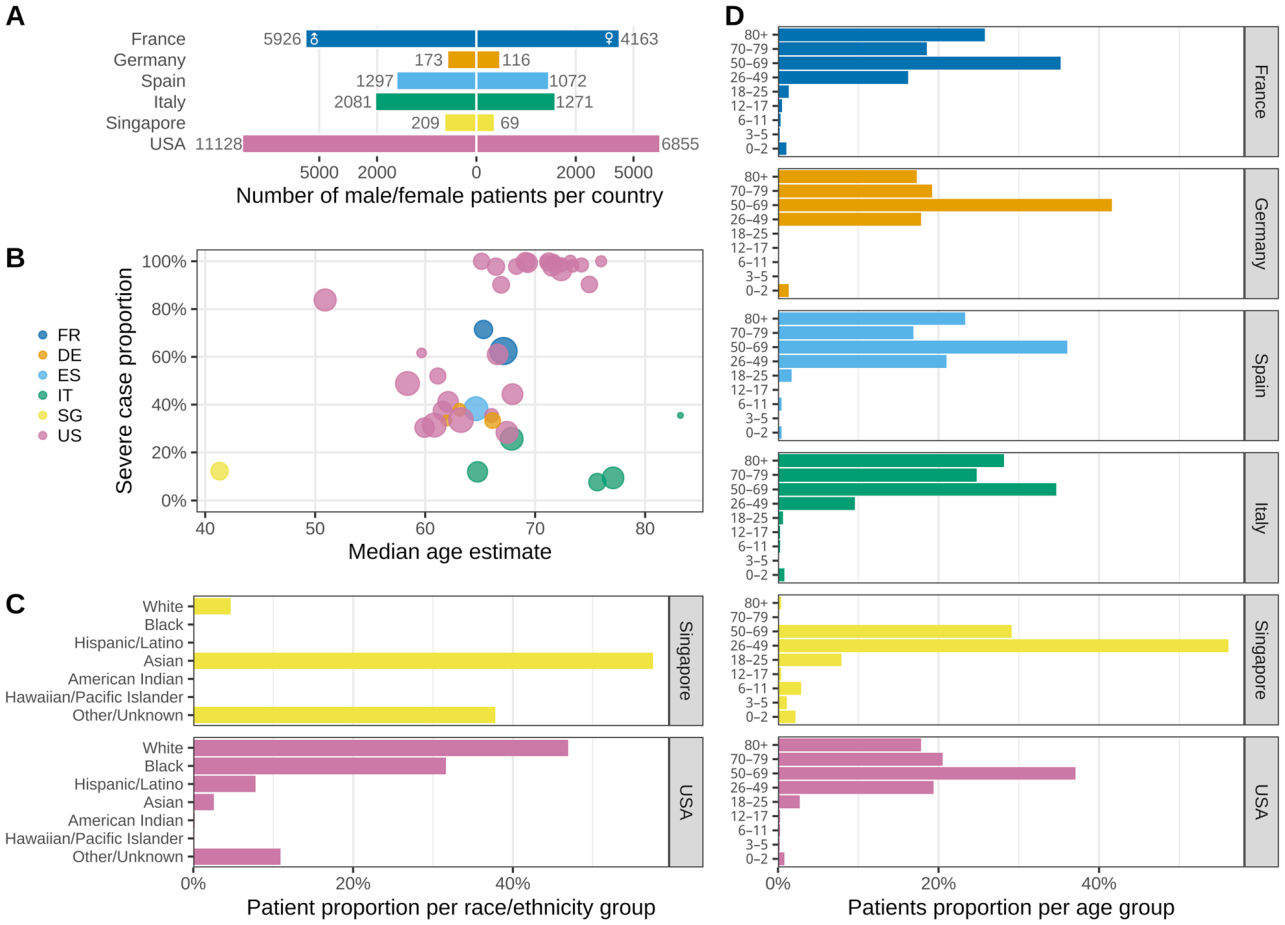
Characteristics of the study population across healthcare systems and countries. (**A**) Total number of male (left) and female (right) patients grouped by country shown in square-root scale. (**B**) Proportion of ever-severe cases by median age estimate at each healthcare system, grouped by country. Node size corresponds to the total number of patients per system. (**C**) Distribution of self-identified race among patients at healthcare systems in Singapore and the United States. The Other/Unknown category includes patients who did not identify with any of the predefined race categories and/or whose data were not reported. Most European healthcare systems did not report race. (**D**) Average proportion of patients in each age group within each country. FR, France; DE, Germany; ES, Spain; IT, Italy; SG, Singapore; US(A), United States of America.

Following a consortium-wide standardized procedure (Fig. 1), we collected the EHR data from 35,177 hospitalized patients with PCR-confirmed SARS-CoV-2 infection from 338 hospitals affiliated with 34 healthcare systems in six countries (eTable 1). Aggregate demographic data were available for 34,647 patients (98.5%). The cohort had a greater proportion of men (20,814, 60.1%) than women (13,546, 39.1%), while 287 (0.8%) patients had unknown gender (Fig. 2A). The study captured a broad range of proportions of severe COVID-19 cases (based on the internationally validated 4CE COVID-19 severity criteria^30^) across this multinational network of healthcare systems. There was no clear relationship between COVID-19 severity and median age (Fig. 2B). Most healthcare systems in Europe did not report race. Among the US healthcare systems, there was a disproportionately high proportion of self-reported Black individuals (Fig. 2C). The study population included a high proportion of individuals above age 50 years and a low proportion of children (age < 18 years) (Fig. 2D).

**Figure 3:**
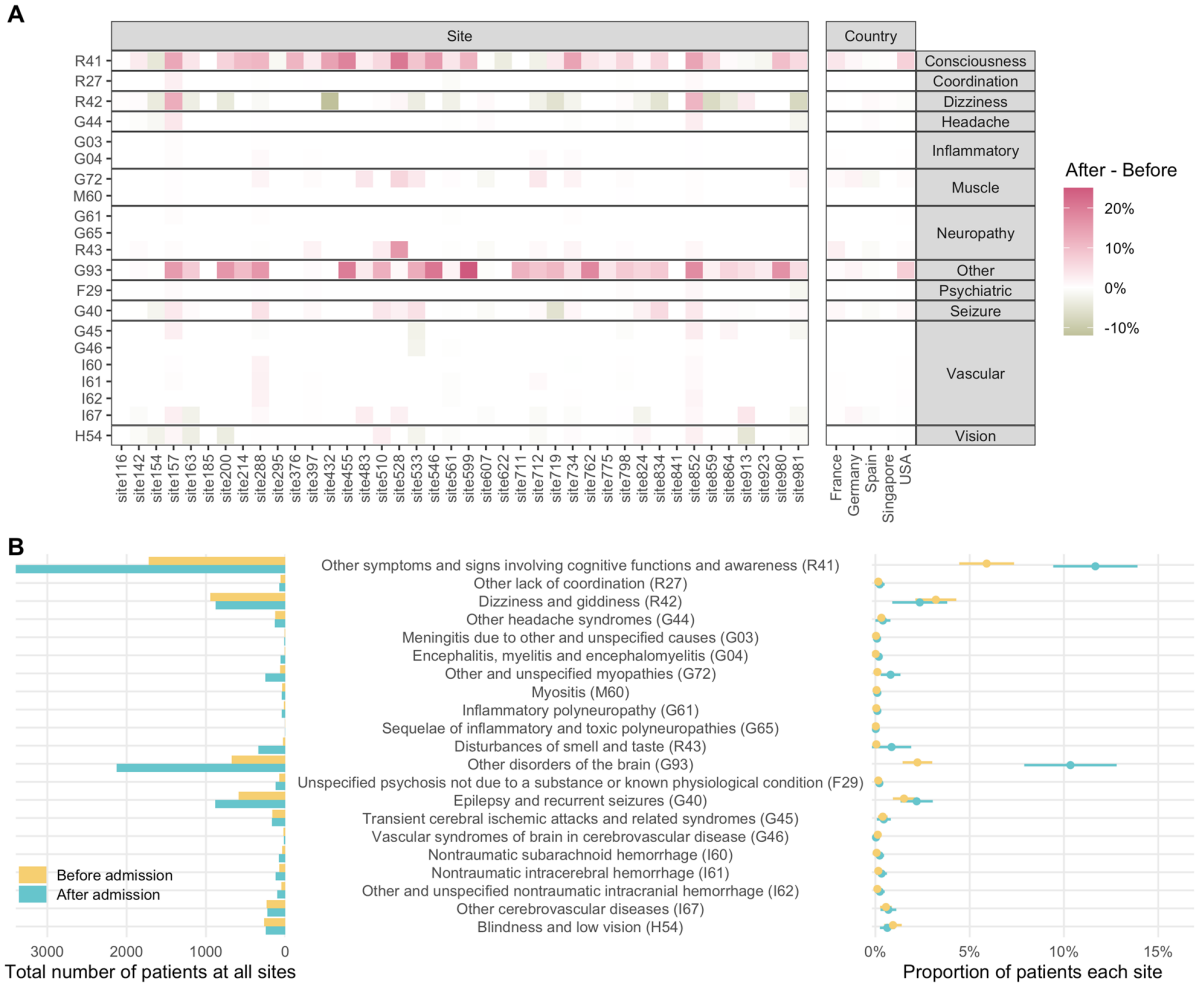
Prevalence of neurological phenotypes among all patients. (**A**) Difference in prevalence of each neurological ICD-10 code by healthcare system and country, calculated as after admission—before admission date (eEq. 2). Pink color on the heat map indicates increased prevalence, while green color indicates decreased prevalence. Please see eFig. 1 for the absolute values of prevalence. (**B**) Total counts of patients with a given neurological ICD-10 code (left) and the mean proportion of patients (right) before and after admission date across all healthcare systems. The mean proportion estimates are shown as circles and the 95% confidence intervals are shown as bars.

We first assessed the prevalence of a wide spectrum of neurological conditions during the first hospital admission for COVID-19, importantly, using the estimates in the 1-year pre-admission period at each healthcare system as the comparator. Towards this end, we used EHR data from the early phase of the pandemic (January to September 2020) and queried all potential neurological conditions based on a comprehensive literature review at the start of the analysis. Most of the contributing healthcare systems (77%) reported an increase in the proportion of hospitalized COVID-19 patients with disorders of consciousness (ICD-10 R41: “Other symptoms and signs involving cognitive functions and awareness”) with a mean increase of 5.8% (95% CI 3.7–7.8%, *p*_FDR_ < 0.001) after admission (Fig. 3, eFig. 1). Similarly, 84% of healthcare systems reported an increase in the proportion of patients with “Other disorders of the brain” (ICD-10 G93, including “encephalopathy”, “cerebral edema”, “brain death”) with a mean increase of 8.1% (5.7–10.5%, *p*_FDR_ < 0.001) after admission (see online interactive data repository: https://covidclinical.github.io/Phase1.1NeuroRCode/01-analysis-icd10.html#prevalence-change-table). The proportion of patients with “epilepsy and recurrent seizures” (ICD-10 G40), “encephalitis, myelitis, and encephalomyelitis” (ICD-10 G04) and “other and unspecified myopathies” (ICD-10 G72) increased after admission, but these findings were not significant after adjusting for multiple testing. Likewise, none of the other neurological conditions showed a statistically significant difference in prevalence after admission.

**Figure 4:**
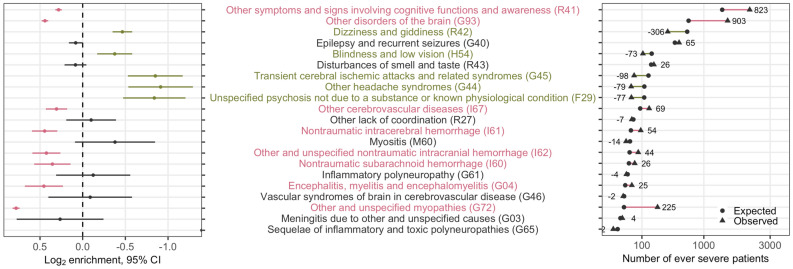
Analysis of enrichment or depletion of neurological conditions after admission in patients with severe disease. For each neurological ICD-10 code, we show the log_2_ enrichment (LOE) and its 95% confidence interval (left), and the absolute difference between the observed (filled triangle) and expected (⋅) number of patients experiencing severe COVID-19 in square-root scale (right). A purple positive LOE value for an ICD-10 code indicates a statistically significantly higher proportion of severe cases having a given neurological ICD-10 code when compared to the never-severe cases. Conversely, a green negative LOE value indicates a statistically significantly lower proportion of severe cases having a given neurological ICD-10 code when compared to the never-severe cases. Neurological ICD-10 codes are ordered by the expected number of severe cases after admission.

To assess the association with COVID-19 severity, we next used Fisher’s exact test to examine the enrichment or depletion of each neurological condition among patients hospitalized for COVID-19 who ever experienced severe disease based on the published 4CE COVID-19 severity criteria^30^, using those who never experienced severe disease as the comparator (Fig. 4). A positive log_2_ value of enrichment (LOE) value denoted a higher proportion of severe cases for a given neurological condition than the never-severe cases, while a negative LOE value indicated the opposite. For instance, a LOE value of 0.283 for ICD code R41 meant that the observed number of severe cases with R41 (disorder of consciousness) was 2^0.283^ or ~ 1.22 times higher than the expected number of severe cases for R41, which was equivalent to a 22% increase in relative risk (*i.e.,* relative risk difference RRD = 22%). Table 1 listed the neurological phenotypes that exhibited *statistically significant* associations with severe COVID-19 status (*p*_FDR_ < 0.05). The interactive data table (https://covidclinical.github.io/Phase1.1NeuroRCode/01-analysis-icd10.html#enrichment_tab) and the results directory of the project online data repository (https://github.com/covidclinical/Phase1.1NeuroRCode/tree/master/results) listed the LOE, 95% confidence intervals and *p* values for *all* neurological conditions examined.

**Table 1:** Statistically significant associations of neurological conditions and severe disease status after admission (*p*_FDR_ < 0.05).

Neurological condition (ICD-10 code)	LOE^a^	RRD^b^ (%)	RRD 95% CI (%)	*p*_FDR_
Blindness and low vision (H54)	− 0.38	− 23	(− 33, − 11)	2.0 × 10^–4^
Dizziness and giddiness (R42)	− 0.47	− 28	(− 33, -22)	6.3 × 10^–19^
Encephalitis, myelitis and encephalomyelitis (G04)	0.45	37	(17, 60)	0.0081
Nontraumatic intracerebral hemorrhage (I61)	0.45	36	(23, 51)	3.6 × 10^–5^
Nontraumatic subarachnoid hemorrhage (I60)	0.35	28	(10, 48)	0.019
Other and unspecified myopathies (G72)	0.78	72	(67, 77)	8.8 × 10^–45^
Other and unspecified nontraumatic intracranial hemorrhage (I62)	0.43	34	(20, 50)	3.0 × 10^–4^
Other cerebrovascular diseases (I67)	0.31	24	(13, 35)	2.0 × 10^–4^
Other disorders of the brain (G93)	0.44	36	(32, 40)	5.6 × 10^–73^
Other headache syndromes (G44)	− 0.91	− 47	(− 59, − 31)	9.4 × 10^–9^
Other symptoms and signs involving cognitive functions and awareness (R41)	0.28	22	(19, 25)	2.1 × 10^–39^
Transient cerebral ischemic attacks and related syndromes (G45)	− 0.85	− 45	(− 56, − 31)	5.0 × 10^–10^
Unspecified psychosis not due to a substance or known physiological condition (F29)	− 0.84	− 44	(− 57, − 28)	7.7 × 10^–8^

^a^The interactive data table (https://covidclinical.github.io/Phase1.1NeuroRCode/01-analysis-icd10.html#enrichment_tab) and the results directory of the project online data repository (https://github.com/covidclinical/Phase1.1NeuroRCode/tree/master/results) show the log_2_ value of enrichment (LOE), 95% confidence intervals, and *p* values for *all* neurological ICD-codes adjusted for multiple hypothesis testing.

^b^RRD: Relative risk difference = Observed relative risk − 1.

In the period after hospital admission for COVID-19, using the patients who never experienced severe COVID-19 as reference, a significantly higher proportion of patients with severe disease had “other symptoms and signs involving cognitive functions and awareness” (ICD-10 R41: RRD_after_ = 22%), “other cerebrovascular disease” (ICD-10 I67: RRD_after_ = 24%), “nontraumatic subarachnoid hemorrhage” (ICD-10 I60: RRD_after_ = 28%), “other and unspecified nontraumatic intracranial hemorrhage” (ICD-10 I62: RRD_after_ = 34%), “nontraumatic intracerebral hemorrhage” (ICD-10 I61: RRD_after_ = 36%), “other disorders of the brain” (ICD-10 G93, including “encephalopathy”: RRD_after_ = 36%), “encephalitis, myelitis and encephalomyelitis” (ICD-10 G04: RRD_after_ = 37%), and “other and unspecified myopathies” (ICD-10 G72, including “inflammatory and immune myopathies” and “critical illness myopathies”: RRD_after_ = 72%) (Table 1, Fig. 4). In contrast, a significantly lower proportion of patients with severe disease had “blindness and low vision” (ICD-10 H54: RRD_after_ = − 23%), “dizziness and giddiness” (ICD-10 R42: RRD_after_ = − 28%), “other headache syndromes” (ICD-10 G44: RRD_after_ = − 47%), “transient cerebral ischemic attacks and related syndromes” (ICD-10 G45: RRD_after_ = − 45%), and “unspecified psychosis not due to a substance or known physiological condition” (ICD-10 F29: RRD_after_ = − 44%) during hospitalization. Subgroup analysis comparing the US and non-US healthcare systems yielded consistent results (eFig. 2).

As 12 healthcare systems in Italy and the US contributed EHR data entirely or partially comprising ICD-9 codes, we performed separate subgroup analyses using only the ICD-9 data (eTable 2), given that one-to-one mapping of some ICD-9 codes to ICD-10 codes was not feasible. To summarize, the prevalence of “disorders of consciousness” and “other neurological conditions” increased after COVID-19 admission date when compared to the 1-year pre-admission period, as in the ICD-10 data analysis (eFigs. 3, 4). There was no statistically significant difference when examining the change in prevalence of other neurological ICD-9 codes after admission date. In severity analysis, there were similarities with the ICD-10 data (*e.g.,* “disorders of consciousness”) but also differences involving seizure and cerebrovascular events that would require caution in interpretation due to differences in sample size (see Supplementary Material, eFig. 5, eTable 3).

## Discussion

Following a standardized approach for aggregating EHR-derived clinical facts from a federated network of multinational healthcare systems while preserving patient privacy, we report the change in prevalence of a wide spectrum of central and peripheral neurological conditions among a large cohort of patients hospitalized with PCR-confirmed SARS-CoV-2 infection across geographically diverse healthcare systems from six countries, using the 1-year period before COVID-19 hospitalization as reference. We further report the difference in the prevalence of neurological conditions among hospitalized patients with severe COVID-19 when compared to the non-severe patients. Given the challenges with evaluating patients and obtaining data during the pandemic, EHR-based studies complement the more labor-intensive physician-reported registry studies.

Disorders of consciousness and other disorders of the brain are the most prevalent neurological phenotypes among all patients hospitalized with COVID-19 during the early pandemic. While the specific underlying causes could be broad (*e.g.,* metabolic disturbance, hypoxia, medication effect, seizures, stroke), these findings are consistent with prior reports and associated with worse health outcomes^19,20,24,25^. Given that hospitalized COVID-19 patients can manifest a wide range of symptoms and signs, certain neurological conditions do not cleanly fall within parent diagnostic codes with clearly specific descriptions (*e.g.,* ICD-10 G04: encephalitis, myelitis and encephalomyelitis; ICD-10 G61: inflammatory polyneuropathy). In the case of “other disorders of brain” (including “unspecified disorders of the brain”), the parent diagnostic code of ICD-10 G93 covers a wide range of conditions, some of which are potentially relevant to hospitalized COVID-19 patients though they do not belong to a single unifying category (*e.g.,* G93.1: anoxic brain damage; G93.2: benign intracranial hypertension; G93.3: post-viral fatigue syndrome; G93.4: other and unspecified encephalopathy; G93.5: compression of brain; G93.6: cerebral edema; G93.82: brain death). Other diagnosis codes under G93 (*e.g.,* G93.9: unspecified disorders of the brain) likely capture symptoms and signs that may *not* be easily categorized, commonly reported (*i.e.,* symptoms and signs that are *not* headache, visual changes, dizziness, confusion, weakness, or sensory changes), and/or revealing any objective pathology based on common clinical tools such as physical exam, laboratory, neuroimaging or electrodiagnostic tests. Here are two potential clinical scenarios that could lead to the diagnosis code of “unspecified disorder of a brain”: (1) non-specific findings on the brain magnetic resonance imaging that were not consistent with ischemic stroke, intracranial hemorrhage, meningitis or encephalitis, neoplasm, or demyelination; (2) subjective sensory complaint without objective findings on exam or diagnostic tests that was not coded with specific diagnosis. In contrast, the diagnosis code ICD-10 R41 (“disorders of consciousness”) specifically refers to symptoms and signs involving awareness and cognitive function, which include disorientation, amnesia, neglect, age-related cognitive decline, altered mental status, specific cognitive deficits involving attention, concentration, communication, visuospatial and psychomotor domains. This code will cover common clinical scenarios in hospitalized patients such as delirium (fluctuating arousal state) and decreased responsiveness.

On the other hand, we find no statistically significant increase in the prevalence of other previously reported neurological conditions such as dizziness, headache, seizure, vascular, and vision disorders after the first admission to the hospital for COVID-19. The lower prevalence of early COVID-19 symptoms such as alterations in smell and taste^13^ in this study is likely attributable to the incomplete documentation of these symptoms in the EHR for the hospitalized patient population, particularly those with severe COVID-19. Crucially, these discrepancies may also be due to methodological differences, as our analysis accounted for baseline pre-admission prevalence by reporting the change in prevalence after admission.

Our other major finding indicates that a significantly higher proportion of hospitalized patients with severe COVID-19 (based on a computational phenotyping algorithm of COVID-19 severity that our group previously published^30^) had disorders of consciousness and other disorders of the brain, encephalitis and/or myelitis, cerebrovascular events and myopathy when compared to patients who never had severe disease. Beyond corroborating prior reports^19,23–25^, our findings highlighted similar patterns across geographically diverse multinational healthcare systems using a standardized approach. First among these findings, disorders of consciousness and other disorders of the brain include altered mental status, disorientation cognitive deficits, and encephalopathy. Hypoxemia from respiratory failure, metabolic disturbance, sedation for advanced respiratory support, acute delirium, and other more specific neurological involvement in the setting of severe COVID-19 could all be contributory^32^.

Second, encephalitis and myelitis have variable manifestations^12^ and were likely under-reported due to difficulties of performing diagnostic studies (*e.g.,* magnetic resonance imaging, lumbar puncture) especially during the early phase of pandemic. Neuropathological examinations have not uncovered evidence of direct viral infection of the central nervous system (CNS)^33^, though more studies are needed to confirm whether the mechanisms underlying encephalitis (with or without myelitis) are direct CNS invasion by SARS-CoV-2, acute systemic inflammation with secondary CNS involvement, and/or post-infectious immune-mediated effect on the CNS^2,34,35^.

Third, our finding of cerebrovascular diseases associated with severe COVID-19 include both ischemic strokes and intracranial hemorrhages (nontraumatic intracerebral and nontraumatic subarachnoid), highlighting the difficult balance when managing severe COVID-19 with respect to antiplatelet and anticoagulation therapy. Strokes that occurred in the setting of COVID-19 were associated with high mortality and morbidity^36^. COVID-19 might increase the risk of ischemic stroke through mechanisms such as activation of innate immune system, cardioembolic events, hypoxia-induced ischemia secondary to severe pulmonary disease, coagulation activation, thrombotic angiopathy and endothelial damage^2^. Proposed mechanisms underlying intracranial hemorrhage in COVID-19 include coagulation abnormalities, endothelial dysfunction, dysregulation of the renin-angiotensin system, and disruption of cerebral blood flow autoregulation^2,37^. Patients with severe COVID-19 may have additional risk factors such as hypertension or cardiovascular disease that could further drive cerebrovascular diseases^38^.

Finally, myopathy is common among severe COVID-19 patients. The cause is likely multifactorial: prolonged or severe critical illness, complications due to multi-organ involvement, or medication-induced myotoxicity (*e.g.,* Hydroxychloroquine, steroids)^2,39^. The current data set does not permit subgroup analysis (*e.g.,* ventilated versus non-ventilated patients). Further, most clinical studies to date, including our own, cannot establish whether the neurological phenotypes associated with severe COVID-19 are the direct consequence of SARS-CoV-9 neurotoxicity or due to secondary causes.

Interestingly, some neurological phenotypes are less prevalent in patients with severe COVID-19, including psychosis, dizziness, vision impairment, transient ischemic attack, and headache. The likely explanation is that critically ill patients with or without respiratory failure would either not have an objective evaluation or proper documentation and coding for these conditions given that patients with severe disease are likely to be sedated and/or having altered mental status.

Several analytical elements strengthen the study. Chief of among them, the federated approach of adopting common EHR data elements and standardized processes for representing clinical events with *local* quality control differentiates this study from other EHR-based efforts, complements physician/neurologist-reported registry efforts^12,24,25,40^ and is well suited for multinational and multi-institutional clinical discovery. Critically, the local clinician experts and data scientists at each 4CE contributing healthcare system ensure the control of EHR data quality according to the consortium standard and improve the study rigor. The overall concordance of the main study findings with other registry-based studies is reassuring. Second, our approach of aggregating EHR data, specifically the ICD codes at categorical level, reduced the potential concern for variations in coding practice across diverse healthcare systems that include general medical, neurology, critical care, and rehabilitation settings. Third, the 4CE contributing healthcare systems shared aggregate clinical data following a pre-defined analysis plan while adhering to multi-national patient privacy laws such as the United States Health Insurance Portability and Accountability Act (HIPAA) and the European Union General Data Protection Regulation (GDPR). Fourth, we rapidly implemented the analysis plan at scale by leveraging existing informatics infrastructures and frameworks at each 4CE contributing healthcare system. This federated approach of using EHR data for clinical discovery presents a complementary and alternative approach to the more labor-intensive registry-based approach. Finally, our prevalence study used the 1-year pre-admission period for comparison, which generated more realistic prevalence estimation of neurological conditions among hospitalized patients with COVID-19 than approaches that do not account for pre-admission prevalence. Given the protracted and changing nature of the COVID-19 pandemic, we intentionally included only data during the first hospitalization for COVID-19, specifically during the early phase of the pandemic, to appropriately account for pre-admission prevalence.

Our study has limitations as the result of trade-offs to standardize common data collection from multinational healthcare systems while strictly preserving patient privacy and adhering to privacy laws governing all contributing healthcare systems. First, this study relied on ICD codes that may not capture fully or accurately the disease phenotypes, particularly for conditions better documented in clinical notes. To standardize the collection of ICD codes across diverse contributing healthcare systems and to mitigate coding discrepancies, we used ICD codes at the categorical level (*e.g.*, the first 3 alphanumeric characters before the decimal point for ICD-10). As such, further characterization of certain conditions such as “other disorders of the brain” was not feasible at this stage, though we are working towards a standardized approach of capturing full ICD codes for the next stage of analyses. Second, because we aggregated data across healthcare systems, we were unable to consolidate all related ICD codes (*e.g.,* organizing into PheCode^41^) at the individual patient level. Similarly, the 4CE consortium is preparing a standardized approach to enable aggregate patient-level analyses from each contributing healthcare system. Third, we might not have captured all pre-admission EHR data if patients did not receive their entire care in the same hospital system as the COVID-19 admission. This is a limitation common to all research using EHR data from countries without universal health systems such as the USA. Reassuringly, subgroup analysis showed consistent results between US and non-US contributing healthcare systems. Finally, contributing healthcare systems with small patient counts used obfuscation to reduce the risk of re-identifying the health systems, but the effect of obfuscation is negligible because few healthcare systems had neurological conditions of interest in cases fewer than the obfuscation level. Despite the limitations of deploying this rapid, scalable, patient privacy-preserving research strategy, our key findings were consistent with prior reports from well-characterized but often smaller, single-country and/or single-center cohort studies.

In conclusion, this multinational prevalence study highlighted a range of central and peripheral neurological phenotypes in hospitalized patients with PCR-confirmed SARS-CoV-2 infection, particularly among patients with severe disease. Our multinational and multi-institutional EHR-based efforts using a standardized procedure and common data elements with careful local data quality control complement registry-based research design. In future studies, we will conduct individual-level analysis using additional EHR data such as complete ICD codes, identify risk factors for worse health outcomes (*e.g.,* hospitalization duration, death, re-admission) and examine long-term sequelae in COVID-19 patients with neurological phenotypes.

## Methods

### Patients and data

4CE contributing healthcare systems began in March 2020 to collect EHR data from hospitalized patients with positive SARS-CoV-2 RT-PCR tests. The analyzed data captured the early phase of the pandemic, spanning from January 2020 through early September 2020. We defined COVID-19-related hospitalization as the first hospital admission that occurred between 7 days before and up to 14 days after the first positive SARS-CoV-2 PCR test. The first admission date within this − 7 to + 14 day window is the index admission date.

According to the 4CE consortium agreement, we de-identified contributing healthcare systems to protect their confidentiality. The institutional review board of each participating health system (eTable 1) approved the sharing of anonymous, aggregate data in compliance with multi-national patient privacy laws exempting the requirement for individual patient consent as there was no direct patient recruitment or contact. Some healthcare systems applied a small level of obfuscation (*i.e.,* masking of low counts, eTable 1) to preserve system-specific privacy and to reduce the risk of patient re-identification, though it had no significant impact on the total patient counts.

Using the 4CE standard of common EHR data elements^28–31^, we collected demographics (age, gender, self-identified race/ethnicity) and the International Classification of Disease (ICD) codes (versions 9 or 10) pertaining to neurological conditions (Fig. 1) as well as COVID-19 severity, based on the internationally validated 4CE COVID-19 severity criteria^30^. Among the contributing healthcare systems, only Italian healthcare systems provided exclusively ICD-9 codes while the rest of the healthcare systems provided predominantly ICD-10 codes. As such, we used ICD-10 data for the main analyses and ICD-9 data for supplementary analyses. To standardize EHR data elements, we used the first three alphanumeric characters of a given ICD code, which designates the category of the disease or injury (*e.g.,* ICD-10 G44 denotes the category of “Other headache syndromes”).

For all patients hospitalized with COVID-19, we collected ICD codes at two time periods, *before* and *after* the date of admission of the first hospitalization for COVID-19. The period before COVID-19 hospitalization ranged from − 365 days to − 15 days preceding the admission date. Inclusion of EHR data up to 1 year before admission is a pragmatic decision to balance available data from all healthcare systems and minimize past medical conditions that might not be relevant. The period after admission date ranged from the date of admission to the end of the hospitalization. According to the pre-planned consortium-wide strategy, we excluded all codes in the 2 weeks preceding the index hospital admission date to ensure that diagnoses before admission were independent of COVID-19. It could take a few days from SARS-CoV-2 infection to symptom onset and additional days before a positive PCR test and/or hospital admission. Similarly, we analyzed ICD codes before and after the admission date according to whether patients ever met the 4CE criteria for severe COVID-19^30^ (Fig. 1).

### Exposures

We first examined all hospitalized patients with PCR-confirmed positive SARS-CoV-2. We then examined hospitalized patients with COVID-19 who met the 4CE criteria for severe COVID-19, including advanced respiratory care management at any point during their hospitalization. Including diagnoses, procedures, laboratory results and medications (Table 2). Because we used aggregate EHR data in this study, patient-level indicators of severity (*e.g.,* patient-level laboratory value or medication) were unavailable. In response, we applied a computational algorithm of COVID-19 severity that the 4CE consortium previously developed and internationally validated (through chart review by *local *clinician experts at participating healthcare systems) to be a clinically reasonable proxy for hospitalized patients who experienced severe COVID-19 status^30^.

**Table 2:** The 4CE criteria of severe COVID-19.

Severe illness category	Clinical events
Diagnoses	Acute respiratory distress syndrome, ventilator-associated pneumonia
Procedures	Insertion of endotracheal tube; invasive mechanical ventilation
Laboratory results	PaCO_2_, PaO_2_
Medications	General anesthetics; benzodiazepine derivatives; muscle relaxants; other hypnotics and sedatives; adrenergic and dopaminergic agents; other cardiac stimulants; other respiratory system products; phosphodiesterase inhibitors; platelet aggregation inhibitors excluding heparin; vasopressin and analogues

Comprising the occurrence of diagnoses, procedures, laboratory results and medications, this computational phenotyping algorithm of severity has been internationally validated (through manual chart review by local clinician experts at participating healthcare systems) to be a clinically reasonable proxy for hospitalized patients who experienced severe status of COVID-19. This approach is applicable when the *aggregate* electronic health records data from each contributing healthcare systems are available but not the patient-level data. Please see Methods and further detail in a separate 4CE publication^30^.

### Neurological outcomes

We queried 21 ICD-10 codes in 12 categories pertaining to neurological phenotypes following a comprehensive literature search in August 2020. The neurological disease categories included consciousness, coordination, dizziness, headache, inflammatory, muscle, neuropathy, psychiatric, seizure, vascular, vision and other unspecified neurological conditions (Table 3).

**Table 3:** Mapping of neurological disease categories to ICD-10 category codes and their descriptions.

Disease category	ICD-10 code^a^	ICD-10 code description
Consciousness	R41	Other symptoms and signs involving cognitive functions and awareness
Coordination	R27	Other lack of coordination
Dizziness	R42	Dizziness and giddiness
Headache	G44	Other headache syndromes
Inflammatory	G03	Meningitis due to other and unspecified causes
Inflammatory	G04	Encephalitis, myelitis and encephalomyelitis
Muscle	G72	Other and unspecified myopathies
Muscle	M60	Myositis
Neuropathy	G61	Inflammatory polyneuropathy
Neuropathy	G65	Sequelae of inflammatory and toxic polyneuropathies
Neuropathy	R43	Disturbances of smell and taste
Other	G93	Other disorders of the brain
Psychiatric	F29	Unspecified psychosis not due to a substance or known physiological condition
Seizure	G40	Epilepsy and recurrent seizures
Vascular	G45	Transient cerebral ischemic attacks and related syndromes
Vascular	G46	Vascular syndromes of brain in cerebrovascular disease
Vascular	I60	Nontraumatic subarachnoid hemorrhage
Vascular	I61	Nontraumatic intracerebral hemorrhage
Vascular	I62	Other and unspecified nontraumatic intracranial hemorrhage
Vascular	I67	Other cerebrovascular diseases
Vision	H54	Blindness and low vision

^a^To standardize the collection of ICD codes across diverse contributing healthcare systems and to mitigate coding discrepancies, we used ICD codes at the categorical level (*e.g.*, the first 3 alphanumeric characters before the decimal point for ICD-10).

### Statistical analyses

We first compared the prevalence of each neurological ICD code and disease category among all hospitalized patients with COVID-19. For each ICD code, we reported the total count and the proportion of patients hospitalized with COVID-19 at each healthcare system (and each country), both before and after admission date (Supplementary Material, eEq. 1). We used the proportion data before admission as reference control. We calculated the difference in the proportion of cases with a given ICD code before and after admission date (eEq. 2) and used paired two-sided t-tests to examine whether there was a statistically significant difference in the proportion after admission date when compared to that before admission date.

We next compared the prevalence of each neurological ICD code and disease category after admission date between patients who ever met the criteria of severe COVID-19 and those who did not, by healthcare system and by country. For each ICD code, we computed the expected number of severe cases (eEq. 3) and compared with the observed number of severe cases for the given neurological code. To examine the difference in proportion of severe cases for the neurological ICD codes, we calculated the enrichment of each neurological ICD code by dividing the observed number of severe cases by the expected number of severe cases and reported a value of log_2_ enrichment (LOE) and its 95% confidence interval (CI) (eEq. 4). We estimated the LOE 95% CI using the Delta method^42^. We chose LOE as a statistic measure for the difference between the proportion of severe cases and never-severe cases for a given neurological condition because it allows symmetric visualization of enrichment or depletion (equivalent confidence intervals for enrichment and depletion. Finally, we computed the *p*-values using Fisher’s exact test^43^ and corrected for multiple hypothesis testing with Benjamini–Hochberg’s false discovery rate (FDR) procedure^44^. A result was statistically significant if *p*_FDR_ < 0.05. All analyses were performed in the R environment.

## Supplementary Information

Supplementary Information.

## Data Availability

The 4CE consortium does not have permission from each individual contributing healthcare system to release electronic health records data for public access. Only aggregate data were shared by healthcare systems for this study. All aggregate data in a de-identified fashion are available for download (http://www.covidclinical.net).

## Code availability

Our analysis results are available in browsable R notebooks (https://github.com/covidclinical/Phase1.1NeuroRCode/) under CC BY 4.0, with source code distributed under a BSD 3-Clause License. Figure 1 was created with Biorender (Biorender.com). Figures 2, 3, 4 as well as all supplementary figures were created using R v.4.0.3 (http://www.R-project.org)^45^ and the package ggplot2^46^.
