# Supplementary material for: Multinational characterization of neurological phenotypes in patients hospitalized with COVID-19
Source: Sci Rep. 2021 Oct 12;11:20238. doi: 10.1038/s41598-021-99481-9 (PMC8510999; doi:10.1038/s41598-021-99481-9)
Supplement: Supplementary file 1 — Supplementary Information. [file 41598_2021_99481_MOESM1_ESM.pdf]

Multinational Characterization of Neurological Phenotypes in Patients Hospitalized with COVID-19

Supplementary Methods 2
Supplementary Results 2-3
eTable 1 4-5
eTable 2 6-7
eTable 3 8
eFigure 1 9
eFigure 2 10
eFigure 3 11
eFigure 4 12
eFigure 5 13

Supplemental Materials

Supplementary Statistical Methods and Equations

For each neurological ICD code (3 alphanumeric characters), we reported the total count across all contributing healthcare systems and all countries (Y) as well as the proportion of patients hospitalized with COVID-19 who had each ICD code at each healthcare system (and each country), both before admission ($prop_{before}$) and after admission ($prop_{after}$) (**eEq. 1**).

$$prop_{before}(X,Y) = \frac{number\ of\ hospitalized\ COVID{-}19\ positive\ cases\ diagnosed\ with\ X\ at\ Y before\ admission}{total\ number\ of\ hospitalized\ COVID{-}19\ positive\ cases\ at\ site\ (or\ country)\ Y}$$ (**eEq. 1a**)

$$prop_{after}(X,Y) = \frac{number\ of\ hospitalized\ COVID{-}19\ positive\ cases\ diagnosed\ with\ X\ at\ Y after\ admission}{total\ number\ of\ hospitalized\ COVID{-}19\ positive\ cases\ at\ site\ (or\ country)\ Y}$$ (**eEq. 1b**)

For each ICD code *X,* we calculated the difference in the proportion of cases with an ICD code *X* before and after COVID-19 hospitalization (**eEq. 2**) and used a paired two-sided t-test to examine whether there was a statistically significant difference when comparing the proportions after admission with proportions before admission across all contributing health systems (and countries).

$$\Delta prop(X,Y) = prop_{after}(X,Y) - prop_{before}(X,Y)$$ (**eEq. 2**)

We next compared the prevalence of each neurological ICD code (first three characters before decimal point) and disease category by contributing healthcare system, before and after admission date, between patients who ever met the 4CE criteria of severe COVID-19 (see main text) and those who did not. For each ICD code *X*, we computed the expected number of ever-severe patient cases s_{Ei} with:

$$s_{Ei} = \frac{m_X}{\sum_{X\in ICD_{neuro}} m_X} \times \sum_{X\in ICD_{neuro}} s_X$$ (**eEq. 3**)

where m_X denotes the observed number of never-severe patient cases with code *X*, and s_X denotes the observed number of ever-severe patient cases with code *X* (**eEq. 3**). Consequently, $\sum_{X\in ICD_{neuro}} s_X$ represents the total number of patients with ever-severe status, and $m_X / \sum_{X\in ICD_{neuro}} m_X$ represents the proportion of patients without severe disease but with neurological code *X*. We then performed an enrichment analysis to examine the difference in proportions of ever-severe disease across neurological ICD codes. Specifically, we calculated the enrichment of each neurological ICD code by dividing the observed number of severe cases by the expected number of severe cases and reported a value of $\log_2$ enrichment (LOE) and its 95% confidence interval (**eEq. 4**).

$$\text{LOE} = log_2 \frac{s_i}{s_{Ei}}$$ (**eEq. 4**)

We estimated the 95% confidence interval of the LOE using the Poisson model method. Finally, we computed the *p* values using Fisher's exact test and corrected for multiple hypothesis testing with Benjamini–Hochberg's false discovery rate (FDR) procedure. We considered a result with $p_{FDR}<0.05$ statistically significant.

Exploratory Analysis of ICD-9 Data

We analyzed separately the neurological phenotypes among the subset of the patients with ICD-9 codes (**eTable 2)**, as a minority of the 4CE contributing healthcare systems in the USA (7 healthcare systems partially submitting ICD-9 codes related to procedure-related ICD-9 codes) and Italy (5 healthcare systems exclusively submitting ICD-9 codes) reported

neurological ICD-9 codes (**eTable 3**). Because 7 of these contributing healthcare systems reported both ICD-9 and ICD-10 data and given the aggregate data format, we could not ascertain the exact number of patients with ICD-9 data. We separated this analysis from the main ICD-10 analysis because one-to-one mapping from ICD-9 to ICD-10 codes was not available for all codes.

As with the ICD-10 data, there was increased prevalence of "disorders of consciousness and other neurological conditions" in patients after admission date when compared to before admission date (**eFig. 2, eFig. 3**). These differences appear to be driven by the contributing healthcare systems in the USA. However, there was no statistically significant difference when examining the change in prevalence of individual neurological conditions after admission date. The smaller sample size might explain the difference from the ICD-10 data results.

Using the patients who never experienced severe disease as the reference control, in the period after admission date, we found a significantly higher proportion of patients with severe disease to have three broad categorical ICD-9 codes (**eFig. 4**): (1) "other conditions of the brain" (ICD-9 348, which broadly includes "cerebral cysts", "anoxic brain damage", "benign intracranial hypertension", "encephalopathy, not elsewhere classified", "compression of brain", "cerebral edema": relative risk difference RRD_{after}=55%); (2) "general symptoms" (ICD-9 780, which broadly includes "altered consciousness", "hallucination", "syncope and collapse", "convulsions", "dizziness and giddiness", "sleep disturbance", "fever and other physiologic disturbances of temperature regulation", "malaise and fatigue": RRD_{after}=33%); (3) "other ill-defined and unknown causes of morbidity and mortality" (ICD-9 799, which broadly includes "asphyxia and hypoxemia", "respiratory arrest", "nervousness", "debility, unspecified", "cachexia", "signs and symptoms involving cognition": RRD_{after}=42%). Given the broad definition of these three categorical ICD-9 codes (348, 780, 799), a direct comparison with the ICD-10 data was not feasible and supported the use of ICD-10 data (over ICD-9 data) in the main analysis. Despite the limitations of these ICD-9 codes, the potential presence of "encephalopathy, not elsewhere classified" (a subcode under ICD-9 348), "altered consciousness" (a subcode under ICD-9 780), and "signs and symptoms involving cognition" (a subcode under ICD-9 799) would be consistent with the main findings from the ICD-10 data. Further, "fever and other physiologic disturbances of temperature regulation", "asphyxia and hypoxemia", and "respiratory arrest" would be consistent with severe COVID-19.

Finally, there was a significantly lower proportion of patients with "other and ill-defined cerebrovascular disease" (ICD-9 437: RDD_{after}=-42%) among patients with severe disease in the period after admission date. However, we must interpret these findings with great caution given the limitations of the ICD-9 data and the inconsistency with the larger sample size of the ICD-10 data.

eTable 1. 4CE network of multinational healthcare systems.

Country	Healthcare System	City	Affiliated Hospitals	Beds	Inpatient discharges/ year	Obfuscation small count mask threshold (e.g., <10)	Obfuscation blurring range if used (e.g., +/-3)
France	Assistance Publique - Hôpitaux de Paris	Paris	39	20,098	1,375,538	<3	none
	Bordeaux University Hospital	Bordeaux	3	2,676	130,033	<3	none
Germany	Erlangen University Hospital	Erlangen	1	1,400	65,000	<5	none
	Medical Center, University of Freiburg	Freiburg	1	1,660	71,500	<4	none
	University Medicine Mannheim	Mannheim	1	1,352	50,748	<3	none
Italy	ICSM Pavia Hospital	Pavia	1	426	8,616	none	none
	ICSM Lumezzane/Brescia Hospitals	Lumezzane/ Brescia	1	149	1,296	none	none
	ICSM Milano Hospital	Milan	1	200	2,432	none	none
	Policlinico di Milano	Milan	1	900	40,000	<5	none
	ASST Papa Giovanni XXIII Bergamo	Bergamo	1	1,080	45,000	<=5	none
	University Magna Graecia of Catanzaro & Mater Domini University Hospital, Catanzaro	Catanzaro	1	210	11,987	none	none
	ASST della provincia di Pavia	Pavia	7	958	29,103	none	none
Singapore	National University Hospital	Singapore	1	1,556	100,977	none	none
Spain	Hospital Universitario 12 de Octubre	Madrid	1	1,256	45,035	<10	none
USA	Beth Israel Deaconess Medical Center	Boston, MA	1	673	40,752	<10	+/-3
	University of Kansas Medical Center	Kansas City, KS	1	794	54,659	<=10	none
	Mass General Brigham (Partners Healthcare)	Boston, MA	10	3,418	163,521	<10	+/-10
	Medical University of South Carolina	Charleston, SC	8	1,600	55,664	<15	none
	University of Pennsylvania	Philadelphia , PA	5	2,469	118,188	<10	none
	University of California, LA	Los Angeles, CA	2	786	40,526	<10	none
	University of Michigan	Ann Arbor, MI	3	1,000	49,008	none	none
	UT Southwestern Medical Center	Dallas, TX	1	608	26,905	<6	none
	Northwestern University	Chicago, IL	10	2,234	103,279	none	none
	University of Pittsburgh / UPMC	Pittsburgh, PA	39	8,085	369,300	none	none
	Medical College of Wisconsin	Milwaukee, WI	3	876	44,655	<11	none
	Wake Forest School of Medicine & Brenner Children's Hospital	Winston Salem, NC	5	1,510	67,541	none	None
	St. Luke's University Health Network	Bethlehem, PA	12	1700	75,000	none	none
	University of Kentucky	Lexington, KY	3	881	45,714	none	none
	Riverside Health System	Newport News, VA	5	670	30,000	none	none

	VA North Atlantic		49	3594	151075	<11	none
	VA Southwest		29	3115	156315	<11	none
	VA Midwest		39	2686	145468	<11	none
	VA Continental		24	2110	113260	<11	none
	VA Pacific		29	2296	114569	<11	none
			**338**	**75,026**	**3,942,664**		

Note: When aggregating the summary statistics at a site, counts below a certain obfuscation threshold (see healthcare system-specific obfuscation parameters) are masked as 0 to preserve system-specific privacy and to reduce the risk of patient re-identification.

eTable 2. Neurological disease categories, corresponding ICD-9 codes, and their descriptions for the exploratory analysis.

Disease Category	ICD-9	ICD-9 Description
Muscle	040	Other bacterial diseases
Psychiatric	298	Other nonorganic psychosis
Headache	307	Special symptoms or syndromes, not elsewhere classified
Inflammatory	320	Bacterial meningitis
Inflammatory	321	Meningitis due to other organisms
Inflammatory	322	Meningitis of unspecified cause
Inflammatory	323	Encephalitis, myelitis, and encephalomyelitis
Other	330	Cerebral degenerations usually manifest in childhood
Other	331	Other cerebral degenerations
Headache	339	Other headache syndromes
Seizure	345	Epilepsy and recurrent seizures
Other	348	Other conditions of brain
Neuropathy	357	Inflammatory and toxic neuropathy
Muscle	359	Muscular dystrophies and other myopathies
Vision	369	Blindness and low vision
Vascular	430	Subarachnoid hemorrhage
Vascular	431	Intracerebral hemorrhage
Vascular	432	Other and unspecified intracranial hemorrhage
Vascular	435	Transient cerebral ischemia
Vascular	436	Acute, but ill-defined cerebrovascular disease
Vascular	437	Other and ill-defined cerebrovascular disease
Muscle	728	Disorders of muscle, ligament, and fascia
Muscle	729	Other disorders of soft tissues
Other	780	General symptoms
Neuropathy	781	Symptoms involving nervous and musculoskeletal systems
Headache	784	Symptoms involving head and neck
Consciousness	797	Senility without mention of psychosis
Consciousness	799	Other ill-defined and unknown causes of morbidity and mortality
Vision/smell/taste	V41	Problems with special senses and other special functions

We note that for the four 3-character neurological ICD-9 codes that did not have a one-to-one mapping to corresponding ICD-10 codes, we manually grouped them into different neurological groups:
V41: Problems with special senses and other special functions (Vision/smell/taste)

437: Other and ill-defined cerebrovascular disease (Vascular)
780: General symptoms (Other)
781: Symptoms involving nervous and musculoskeletal systems (Other)

eTable 3. Statistically significant associations of neurological conditions (ICD-9 codes) after admission and severe disease status ($p_{FDR} < 0.05$).

Neurological Conditions (ICD-9 Code)	LOE [a]	RRD [b] (%)	RRD 95% CI (%)	p_{FDR}
General symptoms (780) [c]	0.41	33	(24, 42)	6.40E-14
Other and ill-defined cerebrovascular disease (437)	-0.76	-41	(-60, -14)	0.022
Other conditions of brain (348)	0.63	55	(37, 74)	1.70E-08
Other ill-defined and unknown causes of morbidity and mortality (799)	0.5	42	(32, 53)	2.70E-15

[a] The results directory of the project online data repository (https://github.com/covidclinical/Phase1.1NeuroRCode/tree/master/results) show the $\log_2$ value of enrichment (LOE), 95% confidence intervals, and *p* values for *all* neurological ICD-codes adjusted for multiple hypothesis testing.
[b] *RRD: Relative Risk Difference = Observed relative risk - 1.*
[c] ICD-9 780 includes “alteration of consciousness”.

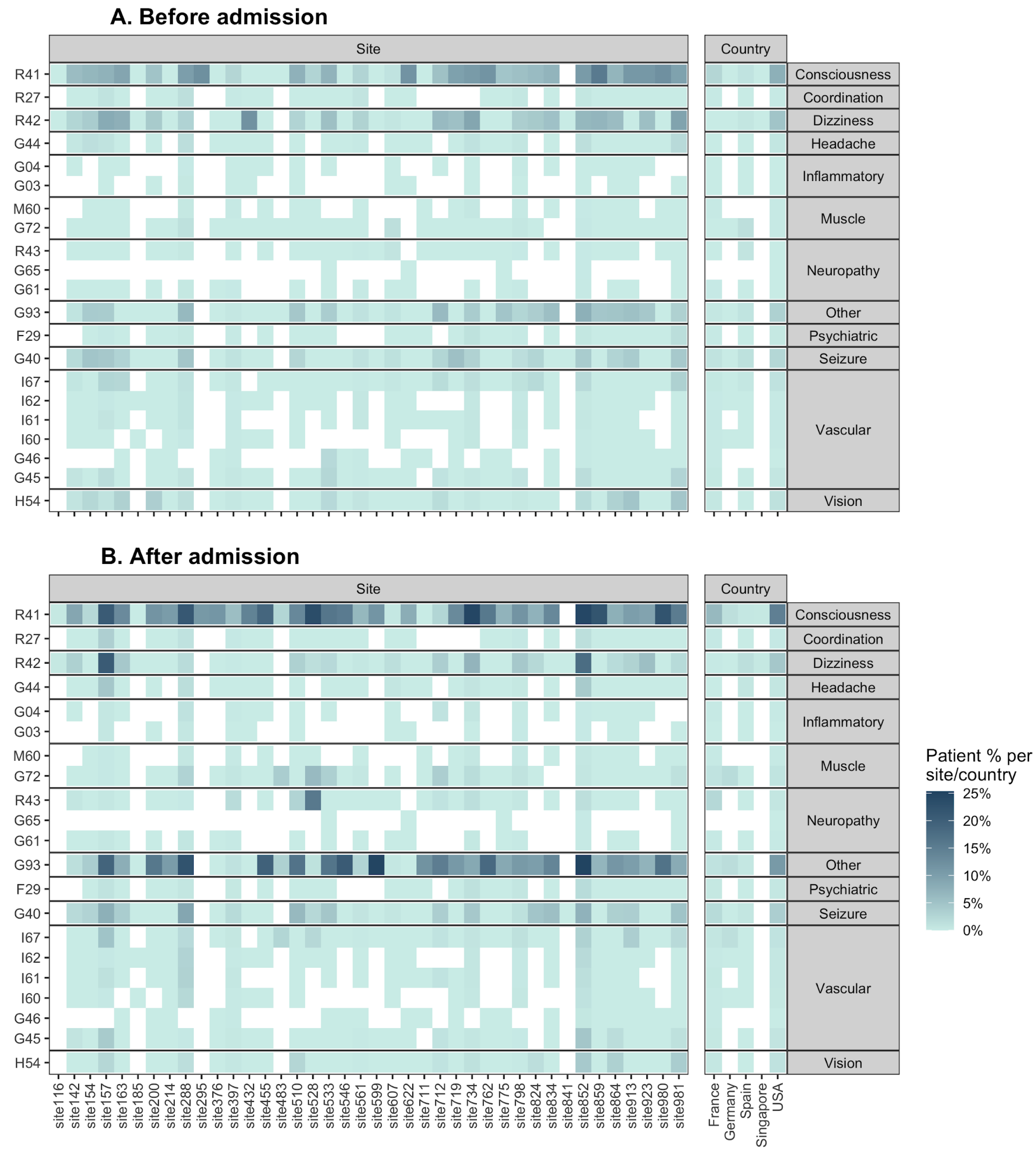

eFigure 1. Prevalence of each ICD-10 code by site and country before and after admission date, supplementing Fig. 3A. Sites refer to the contributing healthcare systems.

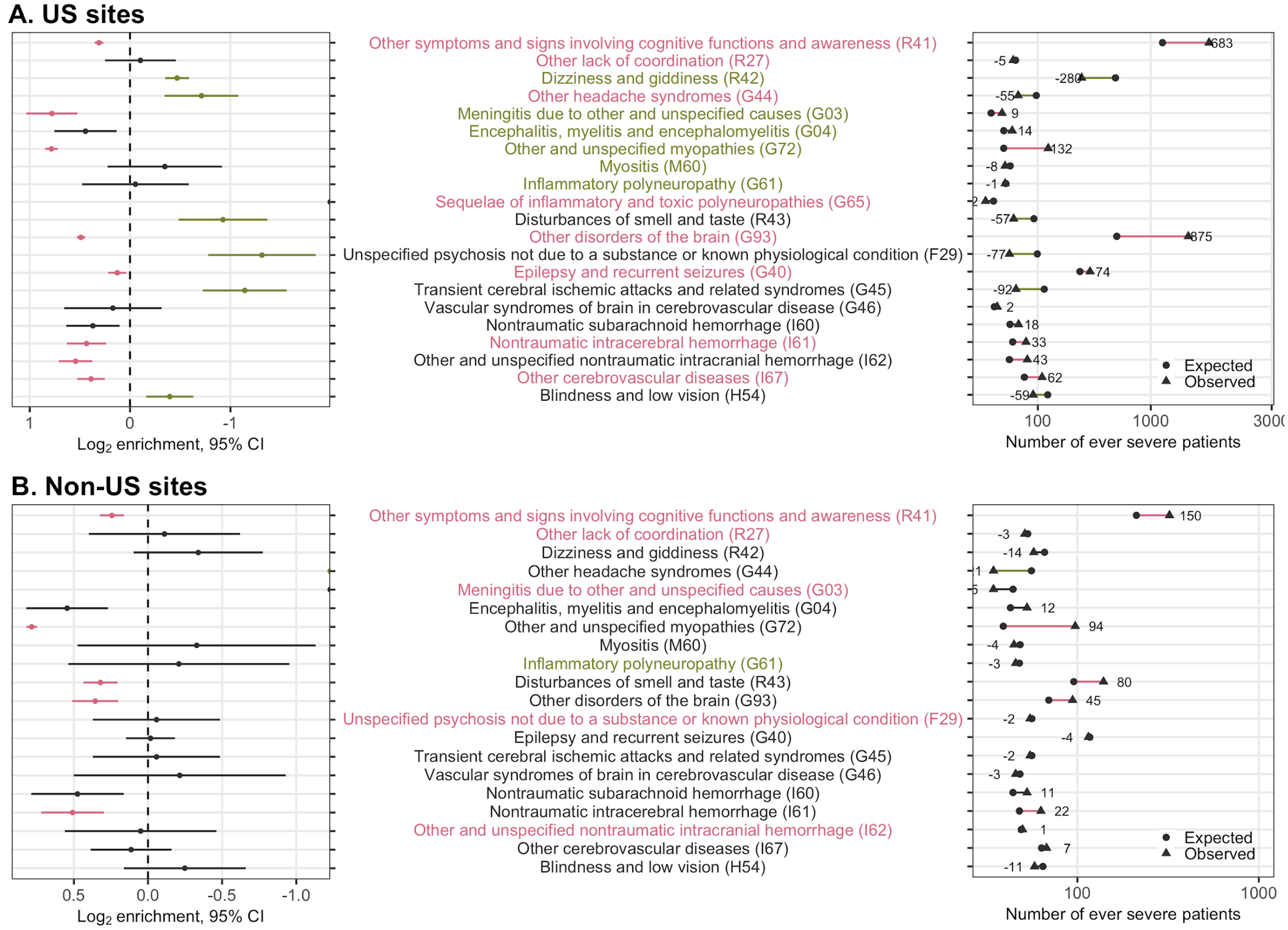

eFigure 2. Prevalence of ICD-10 neurological codes among ever-severe cases, grouped by US sites and non-US sites. Sites refer to the contributing healthcare systems.

Log_2 enrichment (LOE) and 95% confidence interval for each ICD-10 code (left) and the absolute difference between the observed (▲) and expected (●) number of severe cases (right) after admission. A **purple** positive LOE value for an ICD-9 code indicates a statistically significantly higher proportion of severe cases with the given ICD-10 code when compared to the never-severe cases. Conversely, a **green** negative LOE value indicates a statistically significantly lower proportion of severe cases with the given ICD-10 code compared to the never-severe cases. Neurological ICD-10 codes are ordered based on the expected number of severe cases after admission date across all sites. The results are generally consistent between the US sites and the non-US sites, except for the following: (1) ICD-10 code R43 (Disturbances of smell and taste) displays opposite directions between the US sites (higher proportion of severe cases with R43) and the non-US sites (lower proportion of severe cases with R43); (2) The US sites have several significant findings that are not significant among the non-US sites, largely due to the smaller number of sites outside the US. Overall, the findings from the subgroup analyses between US and non-US sites are consistent with the findings from the pooled analysis (**Fig. 4**, main text).

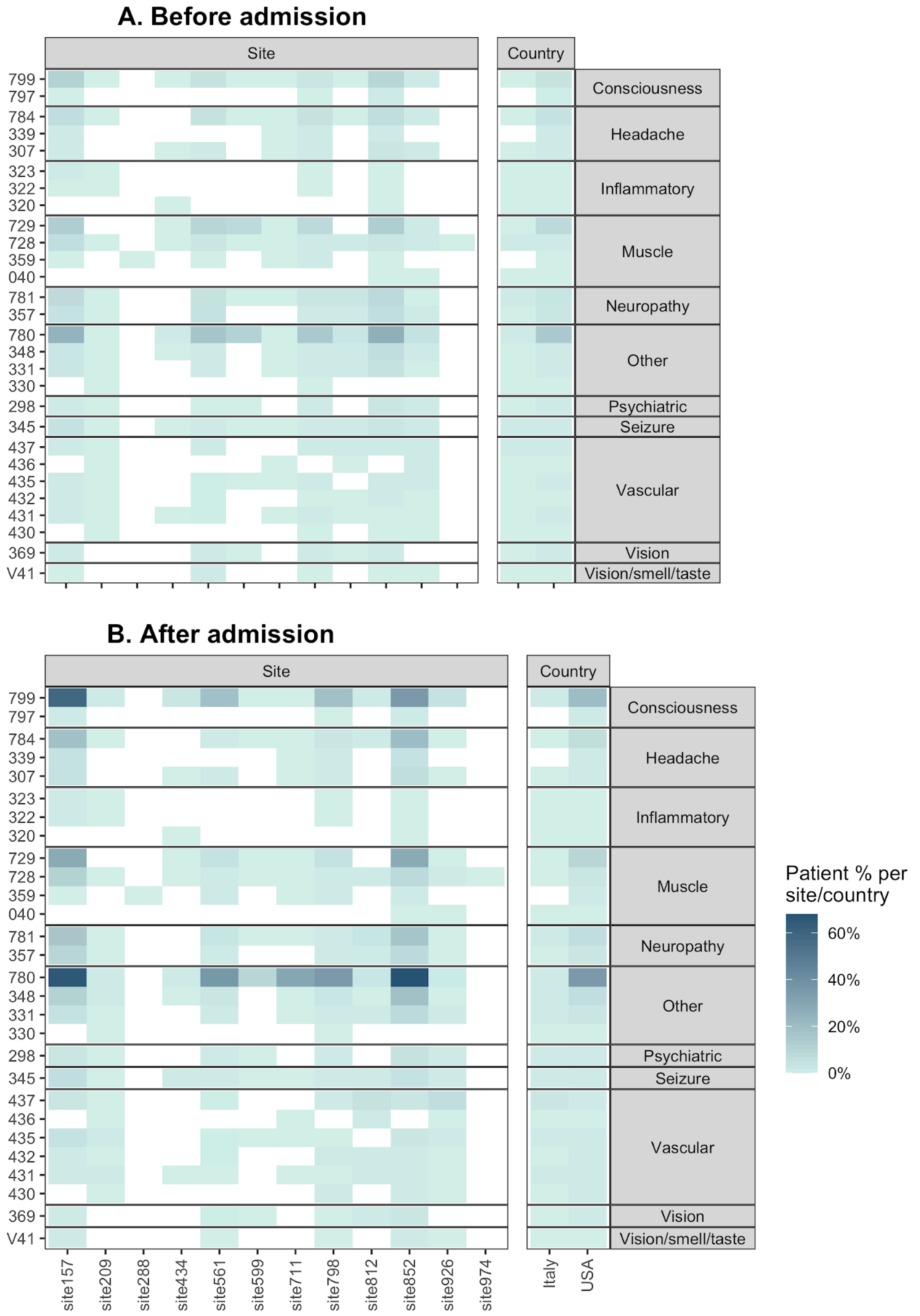

eFigure 3. Prevalence of each ICD-9 code by site and country before and after admission date. Sites refer to the contributing healthcare systems.

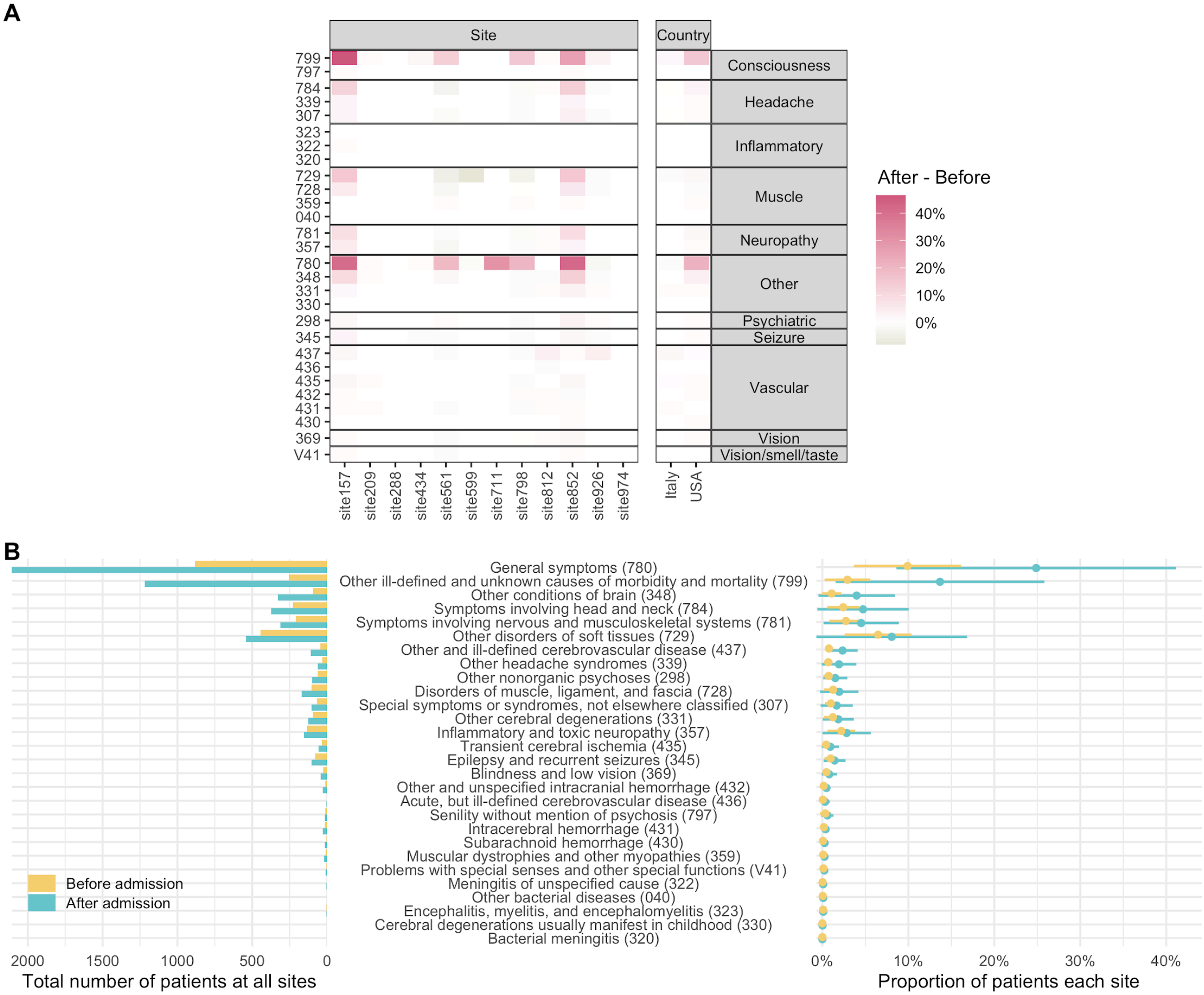

eFigure 4. Prevalence of neurological phenotypes among all patients by ICD-9 code.

Sites refer to the contributing healthcare systems.

(A) Difference in prevalence of each neurological ICD-9 code by site and country, calculated as after admission date - before admission date (Eq. 2). The absolute values of prevalence are displayed in **eFig. 3**. (B) Per ICD-9 code, total counts of patients at all sites (left) and average proportion of patients (right) before and after admission date. Mean prevalence estimates across sites are shown as circles and their 95% confidence intervals as bars. ICD-9 codes are ordered based on the mean prevalence difference between before and after admission date.

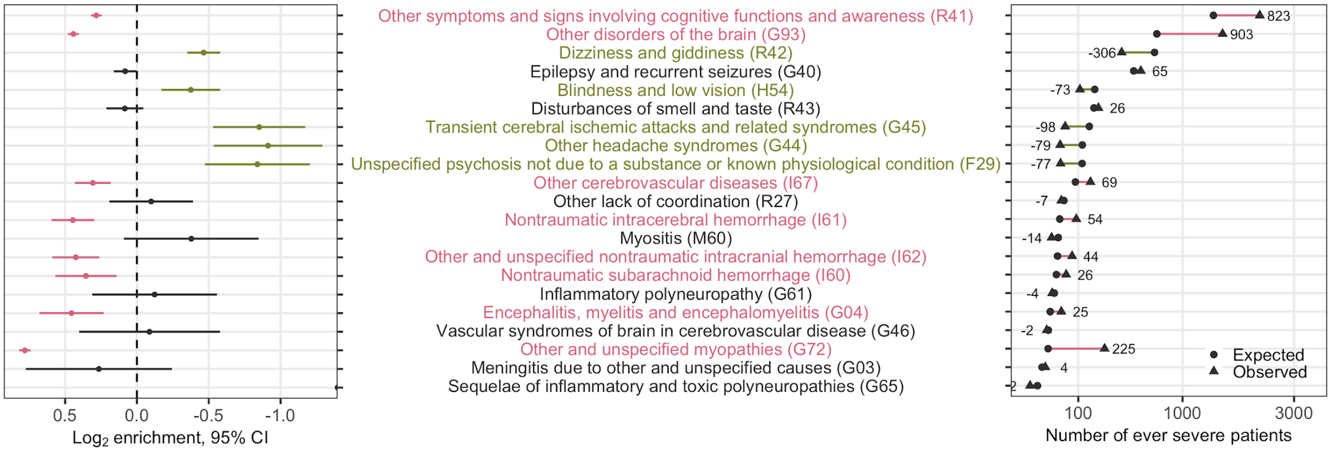

eFigure 5. Prevalence of ICD-9 neurological codes among ever-severe cases. Sites refer to the contributing healthcare systems.

Log_2 enrichment (LOE) and 95% confidence interval for each ICD-9 code (left) and the absolute difference between the observed (▲) and expected (●) number of severe cases (right) after admission. A **purple** positive LOE value for an ICD-9 code indicates a statistically significantly higher proportion of severe cases with the given ICD-9 code when compared to the never-severe cases. Conversely, a **green** negative LOE value indicates a statistically significantly lower proportion of severe cases with the given ICD-9 code compared to the never-severe cases. Neurological ICD-9 codes are ordered based on the expected number of severe cases after admission date.
